# High prevalence of extended-spectrum beta-lactamase-producing *Escherichia coli* and *Klebsiella pneumoniae* isolates: A 5-year retrospective study at a Tertiary Hospital in Northern Thailand

**DOI:** 10.3389/fcimb.2022.955774

**Published:** 2022-08-08

**Authors:** Achiraya Siriphap, Thawatchai Kitti, Akachai Khuekankaew, Chalermchai Boonlao, Chonthida Thephinlap, Chutamas Thepmalee, Nittiya Suwannasom, Krissana Khoothiam

**Affiliations:** ^1^ Division of Microbiology, School of Medical Sciences, University of Phayao, Phayao, Thailand; ^2^ Faculty of Oriental Medicine, Chiang Rai College, Chiang Rai, Thailand; ^3^ Department of Clinical Microbiology, Chiangrai Prachanukroh Hospital, Chiang Rai, Thailand; ^4^ Division of Biochemistry, School of Medical Sciences, University of Phayao, Phayao, Thailand

**Keywords:** prevalence, ESBL, *Escherichia coli*, *Klebsiella pneumoniae*, antibiotic resistance

## Abstract

**Background:**

The global emergence and spread of extended-spectrum beta-lactamase (ESBL)-producing *Enterobacterales*, especially *Escherichia coli* and *Klebsiella pneumoniae*, have been recognized as a public health concern as severe infections caused by these microorganisms increase morbidity and mortality. This study aimed to assess the prevalence of ESBL-positive *E. coli* and *K. pneumoniae* strains isolated from hospitalized patients in Chiangrai Prachanukroh hospital, Chiangrai province, Thailand.

**Methods:**

This retrospective analysis was conducted from January 2016 to December 2020. A total of 384,001 clinical specimens were collected aseptically and further cultivated on an appropriate medium. All clinical isolates (one isolate per patient) were identified based on standard laboratory methods. Antibiotic susceptibility testing was performed by the Kirby Bauer disc diffusion technique following CLSI guidelines. ESBL production was screened with ceftazidime and cefotaxime discs based on the CLSI recommendations. Phenotypic confirmation of ESBL production was carried out using a double-disc synergy technique following the CLSI standard.

**Results:**

Of a total of 384,001 clinical samples analyzed for bacterial species identification, 11,065 (2.9%) tested positive for *E. coli* and 5,617 (1.5%) for *K. pneumoniae*. Approximately 42.5% (4,706/11,065) of *E. coli* and 30.2% (1,697/5,617) of *K. pneumoniae* isolates were classified as ESBL producers. A higher proportion of ESBL producers was found in patients older than 60 years and male groups. The highest infection rates of ESBL-positive pathogens were observed among patients in a medical unit. ESBL-producing *E. coli* and *K. pneumoniae* isolates were predominantly found in urine and sputum, respectively. ESBL producers exhibited a high resistance rate to ampicillin (99.8–100%), cefazolin (100%), cefotaxime (100%), fluoroquinolones, and trimethoprim/sulfamethoxazole.

**Conclusions:**

This study demonstrated the high prevalence and emerging antibiotic resistance of ESBL-positive *E. coli* and *K. pneumoniae* isolates from patients admitted to a provincial hospital in northern Thailand. Most ESBL-producing strains were highly resistant to several antimicrobial agents apart from carbapenems and aminoglycosides. These findings indicated that carbapenems and aminoglycosides should be advised as the first-line drugs of choice for serious infections with ESBL-producing *Enterobacterales*.

## Introduction

Antimicrobial resistance has risen worldwide and contributes to severe morbidity and mortality in settings with limited diagnostic and healthcare (Schwaber and Carmeli, 2007; Founou et al., 2017). The prevalence of extended-spectrum beta-lactamase (ESBL)-producing *Enterobacterales* has increased steadily worldwide, and these bacteria are recognized as a significant source of antibiotic resistance among Gram-negative bacteria (Coudron et al., 1997; Schwaber and Carmeli, 2007; Bush and Fisher, 2011; Murray and Peaper, 2015). *Enterobacterales*, particularly *Escherichia coli* and *Klebsiella pneumoniae*, are the primary opportunistic agents that play significant roles in hospitalized patients, causing bacteremia as well as urinary and respiratory tract infections (Kim et al., 2002; Khanfar et al., 2009). *E. coli* and *K. pneumoniae* are currently considered the two most common ESBL-producing pathogens in the hospital setting (Obeng-Nkrumah et al., 2013; Ouedraogo et al., 2016). ESBL-producing bacteria are capable of hydrolyzing third-generation cephalosporins and monobactams, but their activity is inhibited by clavulanic acid and tazobactam (Rawat and Nair, 2010). ESBLs are encoded by genes found on large plasmids that share genes for antimicrobial resistance with other pathogens. ESBLs are frequently transmitted by plasmids and can thereby be distributed among hospitalized patients, driving their spread across regions (Paterson and Bonomo, 2005; Paterson, 2006). The increasing emergence of ESBL-producing pathogens has been documented worldwide and varies among countries (Quan et al., 2017; Abayneh et al., 2018; Mineau et al., 2018; Kettani Halabi et al., 2021). In Thailand, the prevalence of ESBL-producing microorganisms in asymptomatic individuals has been recorded, with incidences ranging from 13.0% to 31.2% (Kiratisin et al., 2008; Hongsuwan et al., 2014; Sawatwong et al., 2019). However, representative studies on the emergence of ESBL producers (*E. coli* and *K. pneumoniae*) and trends in antibiotic susceptibility in Southeast Asia are currently limited. Therefore, it is essential to understand the prevalence and epidemiological features of antimicrobial resistance in this geographic region. This study aimed to assess the prevalence of ESBL-positive *E. coli* and *K. pneumoniae* isolated from microbiological samples from hospitalized patients at a provincial hospital in northern Thailand.

## Materials and methods

### Study setting and data collection

This retrospective descriptive study was performed over 5 years between January 2016 and December 2020 in Chiangrai Prachanukroh Hospital, a 758-bed tertiary hospital in the north of Thailand. This study was approved by the Human Ethics Committee of the University of Phayao (ethical approval number 1.1/037/63). Clinical data of individuals visiting the hospital were collected through computerized medical records and clinical chart reviews. The following data were obtained from medical records: age, gender, admission time, hospital unit of admission during infection, causative microorganisms (only the first isolates of bacterial species from each patient), specimen type, and antibiotic susceptibility profile.

### Identification of isolates

Clinical specimens, including blood, urine, sputum, pus, and body fluids as per the hospital records, were cultivated on MacConkey agar plates and incubated aerobically at 37°C for 18–24 hours. Lactose-fermenting strains, which presented as pink colonies on the agar plates, were isolated. The clinical isolates were further identified as *E. coli* or *K. pneumoniae* depending on their morphology in Gram’s staining, bacterial cultures, and biochemical characteristics, as described previously (Jorgensen et al., 2015).

### Antibiotic susceptibility testing

Antibiotic susceptibility testing was performed by the disk diffusion technique on Mueller Hinton Agar (MHA) plates following the Clinical Laboratory Standards Institute (CLSI) guidelines (Clinical and Laboratory Standards Institute, 2014). Three to four colonies were transferred into tubes containing sterile saline, and the samples were then adjusted to obtain the 0.5 McFarland turbidity standard. The bacterial suspensions were homogeneously spread on MHA agar plates with a sterile cotton swab. Antimicrobial discs were then placed on the plates. The antibiotics tested in this work include ampicillin (30 µg), amoxicillin/clavulanic acid (20/10 µg), piperacillin/tazobactam (100/10 µg), cefazolin (30 µg), cefoperazone/sulbactam (30/15 µg), cefotaxime (30 µg), ceftazidime (30 µg), ertapenem (10 µg), imipenem (10 µg), meropenem (10 µg), ciprofloxacin (5 µg), norfloxacin (10 µg), amikacin (30 µg), gentamicin (10 µg), and trimethoprim/sulfamethoxazole (1.25/23.75 µg).

### Determination of ESBL Producers

Screening for ESBL production was conducted by two single disk diffusion tests with ceftazidime and cefotaxime. The positive results were defined as zones of inhibition of ≤ 22 mm or ≤ 27 mm for ceftazidime and cefotaxime, respectively. Confirmatory testing was performed using a combination disk test following the CLSI guideline (Clinical and Laboratory Standards Institute, 2014). Briefly, a disk containing cephalosporin alone (ceftazidime or cefotaxime) or in the presence of clavulanic acid was placed on MHA agar plates at a length of 20 mm (center to center). The test was considered a positive ESBL result when an increase in inhibition zone diameter (over 5 mm) of a disk containing cephalosporin plus clavulanic acid compared to a disk containing cephalosporin alone.

### Data analysis

The data were entered into Microsoft Excel 2016. Data were analyzed using descriptive statistics and presented as frequencies and percentages. The chi-square test was used for analyzing the relationship between categorical variables. Analyses were performed using GraphPad Prism (version 5.00 for Windows). A *P-value* less than 0.05 was considered statistically significant.

## Results

### Prevalence of ESBL-producing *E. coli* and *K. pneumoniae* isolates

A total of 384,001 clinical specimens were tested during the study period (blood: 212,522; urine: 70,759; sputum: 63,335; pus: 20,304; and body fluids: 17,081). A total of 11,065 (2.9%) *E. coli* and 5,617 (1.5%) *K. pneumoniae* isolates were obtained from the samples, of which 4,706/11,065 (42.5%) and 1,697/5,617 (30.2%) were ESBL-producing *E. coli* and *K. pneumoniae* strains, respectively, as seen in **Table 1**. Additionally, the prevalence of ESBL-producing *E. coli* isolates decreased significantly over the study period, from 46.2% in 2016 to 40.9% in 2020 (*P* < 0.05). As for *K. pneumoniae*, the frequency of ESBL-positive strains varied slightly over the 5-year period, ranging from 35.7% in 2017 to 27.6% in 2020 (**Table 1**).

**Table 1:** The prevalence of ESBL-producing *E. coli* and *K. pneumoniae* isolates per year (between 2016 and 2020).

Microorganisms	ESBL-positive isolates/total species (%)						*P* -value
	2016	2017	2018	2019	2020	Total	
*E. coli*	331/716 (46.2)	1191/2707 (44.0)	1021/2544 (40.1)	1164/2658 (43.8)	999/2440 (40.9)	4,706/11,065 (42.5)	0.0023
*K. pneumoniae*	116/494 (33.6)	441/1153 (35.7)	366/1310 (27.9)	404/1390 (29.1)	350/1270 (27.6)	1,697/5,617 (30.2)	0.199

### Epidemiological characteristics of hospitalized patients infected with ESBL producers

Bacterial isolates were predominantly detected in men, with a higher proportion of ESBL-producing *E. coli*, and *K. pneumoniae* isolated from male patients (*E. coli*: 51.1%; *P*<0.001, *K. pneumoniae* 62.1% with no significance) than in female groups (**Table 2**). The age distribution revealed that the maximum number of ESBL producers (*E. coli* and *K. pneumoniae*) was seen in patients over 60 years old (57.0%; *P*<0.001 and 56.0%with no significance, respectively). Patients in the ESBL- and non-ESBL-positive groups were further categorized by hospital unit. The prevalence of ESBL producers was significantly (*P*<0.001) high among isolates from individuals admitted to medical units (*E. coli*: 46.0%, *K. pneumoniae*: 40.4%) (**Table 2** **)**. Among the various clinical specimens analyzed, the frequency of ESBL-positive *E. coli* isolates was highest in urine (53.8% with no significance) while ESBL-positive *K. pneumoniae* strains were predominantly detected in sputum samples (44.2%; *P*<0.001), as seen in **(** **Table 2** **)**.

**Table 2:** Clinical characteristics of patients infected with ESBL-producing and non-ESBL-producing *E. coli* and *K. pneumoniae* isolates.

	Total	*E. coli*		*P-value*	Total	*K. pneumoniae*		*P-value*
Parameters	*E. coli* isolates	ESBL negative (%)	ESBLpositive (%)		*K. pneumoniae*isolates	ESBL negative (%)	ESBL positive (%)	
	n = 11,065	n = 6,359	n = 4,706		n = 5,617	n = 3,920	n = 1,697	
**Gender**								
Male	5,168	2,762 (43.4)	2,406 (51.1)	< 0.001^a^	3,439	2,380 (60.7)	1,059 (62.4)	0.244^a^
Female	5,897	3,597 (56.6)	2,300 (48.9)		2,178	1,540 (39.3)	638 (37.6)	
**Ages**								
<60	4,838	2,815 (44.3)	2,023 (43.0)	<0.001^a^		1,690 (43.1)	746 (44.0)	0.575^a^
< 15	520	345 (5.4)	175 (3.7)		226	112 (2.9)	114 (6.7)	
16-30	477	264 (4.2)	213 (4.5)		231	156 (3.9)	75 (4.4)	
31-45	1,053	604 (9.5)	449 (9.5)		530	390 (10.0)	140 (8.3)	
46-60	2,788	1,602 (25.2)	1,186 (25.2)		1,449	1,032 (26.3)	417 (24.6)	
> 60	6,227	3,544 (55.7)	2,683 (57.0)		3,181	2,230 (56.9)	951 (56.0)	
**Hospital units**								
Medicine	6,504	4,339 (68.2)	2,165 (46.0)	< 0.001^b^	3,053	2,367 (60.4)	686 (40.4)	< 0.001^b^
Surgery	3,028	958 (15.1)	2,070 (44.0)	< 0.001^b^	1,136	518 (13.2)	618 (36.4)	< 0.001^b^
Intensive Care Units	775	527 (8.3)	248 (5.3)	< 0.001^b^	1,094	783 (20)	311 (18.3)	0.1629^b^
Obstetrics and Gynecology	51	23 (0.4)	28 (0.6)	0.099^b^	10	7 (0.2)	3 (0.2)	0.7414^b^
Pediatrics	474	348 (5.5)	126 (2.7)	< 0.001^b^	136	89 (2.3)	47 (2.8)	0.3063^b^
Ear eye nose throat	21	19 (0.3)	2 (0.04)	0.004^b^	82	74 (1.9)	8 (0.5)	< 0.0001^b^
Other^c^	212	145 (2.3)	67 (1.4)	0.001^b^	106	82 (2.1)	24 (1.4)	0.1081^b^
**Specimen types**								
Urine	5,834	3,304 (52.0)	2,530 (53.8)	0.063^b^	1,346	891 (22.7)	455 (26.8)	0.0011^b^
Sputum	593	260 (4.1)	333 (7.1)	< 0.001^b^	2,088	1,338 (34.1)	750 (44.2)	< 0.0001^b^
Blood	2,519	1,557 (24.5)	962 (20.4)	< 0.001^b^	1,154	947 (24.2)	207 (12.2)	< 0.0001^b^
Pus	1,830	1,096 (17.2)	734 (15.6)	0.023^b^	836	624 (15.9)	212 (12.5)	0.0011^b^
Body fluids	289	142 (2.2)	147 (3.1)	0.004^b^	193	120 (3.1)	73 (4.3)	0.0236^b^

Note that; ^a^: *P-value* calculated by the chi-square test provides to evaluate the existence of a link between ESBL-positive isolates and clinical characteristics (gender and ages). ^b^: The *P-value* for differences in various categories (hospital units and specimen types) between ESBL-producing and non-ESBL-producing isolates. ^c^: Hemodialysis and kidney dialysis units.

### Antibiotic resistance profiles

**Figure 1:**
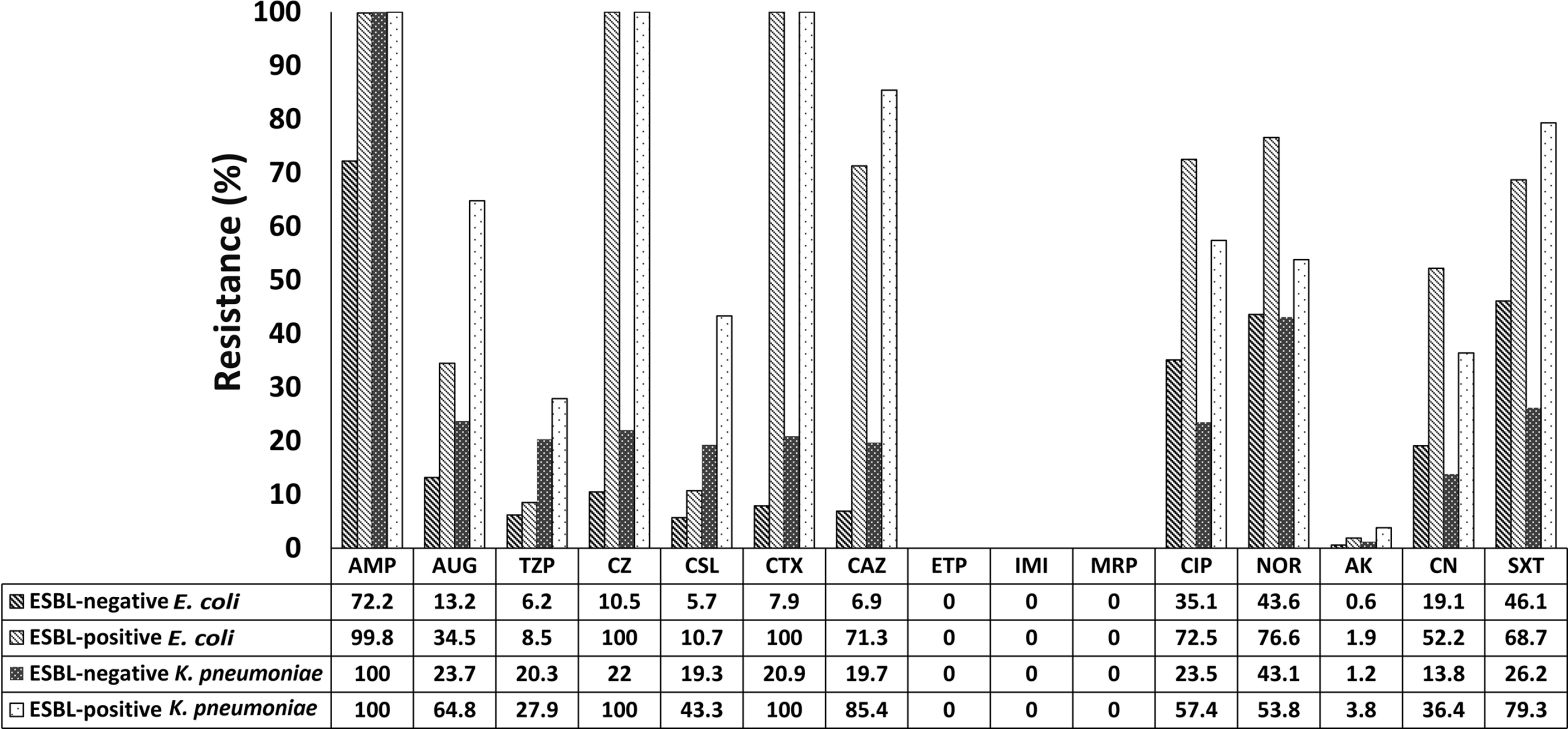
Antibiotic resistance profiles of ESBL-producing and non-ESBL-producing *E. coli* and *K. pneumoniae* isolated from patients admitted to Prachanukroh hospital, Chiangrai, Thailand (2016-2020). AMP, Ampicillin; AUG, Amoxicillin/Clavulanic acid; TZP, Piperacillin/Tazobactam; CZ, Cefazolin; CSL, Cefoperazone/Sulbactam; CTX, Cefotaxime; CAZ, Ceftazidime; ETP, Ertapenem; IMI, Imipenem; MRP, Meropenem; CIP, Ciprofloxacin; NOR, Norfloxacin; AK, Amikacin; CN, Gentamicin; SXT, Trimethoprim/Sulfamethoxazole.

Antibiotic resistance patterns differed between ESBL-producing and non-ESBL-producing isolates. Overall, ESBL-positive *E. coli* strains had higher resistance rates than non-ESBL-producing *E. coli* to ceftazidime (71.3% versus 6.9%), ciprofloxacin (72.5% versus 35.1%), and trimethoprim/sulfamethoxazole (68.7% versus 46.1%). At the same time, ESBL-producing *K. pneumoniae* strains exhibited high resistance rates to these antibiotics as follows: ceftazidime (85.4%), ciprofloxacin (57.4%), and trimethoprim/sulfamethoxazole (79.3%). Notably, all ESBL-producing isolates were susceptible to the carbapenem group of antibiotics (imipenem, ertapenem, and meropenem; **Figure 1**).

Regarding aminoglycosides, a minority of ESBL producers showed resistance to amikacin (1.9% and 3.8% of *E. coli* and *K. pneumoniae*, respectively; **Figure 1**), while an increase in resistance to gentamycin was observed (*E. coli*: 52.2%; *K. pneumoniae*: 36.4%). Approximately 8.5% and 10.7% of ESBL-producing *E. coli* strains were resistant to piperacillin/tazobactam and cefoperazone/sulbactam, respectively; the corresponding figures for *K. pneumoniae* were 27.9% and 43.3%, respectively (**Figure 1**).

## Discussion

Recently, infections caused by ESBL-producing *Enterobacterales*, especially *E. coli* and *K. pneumoniae*, were recognized globally as a public health problem (Pitout and Laupland, 2008; Liebana et al., 2012). The emergence of ESBL-producing isolates among clinical specimens is changing over time and varies worldwide and across geographic regions. Notably, the incidence of colonization and infection rates of ESBL-producing pathogens were shown to be up to 25% in several hospitals in Thailand (Hongsuwan et al., 2014; Sawatwong et al., 2019). Therefore, it is necessary to gain knowledge of the acquisition and transmission of ESBL-producing microorganisms in different regions. Our study reported on the prevalence of ESBL-positive *E. coli* and *K. pneumoniae* isolates in clinical samples from individuals admitted to a provincial hospital in the north of Thailand between 2016 and 2020. In this study, the prevalence of ESBL-producing *Enterobacterales* (*E. coli* and *K. pneumoniae*) was 38.4% (6,403/16,682; **Table 1**), which was high compared to that documented by Hongsuwan et al. (2014; 31.2%), Kiratisin et al. (2008; 13.0%), and Sawatwong et al. (2019; 27.0%), in different regions of Thailand. However, the observed prevalence was low compared to the results of an investigation in Vietnam, which documented an ESBL producer prevalence of up to 55.1% (Suwantarat and Carroll, 2016). Additionally, the infection rates of ESBL-producing *Enterobacterales* in Southeast Asian countries varied widely, being 19.8% in Singapore, 36.8% in the Philippines, and 40.6% in Cambodia (Suwantarat and Carroll, 2016; Caron et al., 2018). The identified ESBL-producers were predominantly *E. coli* strains compared to *K. pneumoniae*, similar to the findings of previous studies (Kiratisin et al., 2008; Quan et al., 2017).

As noted in previous reports, patient gender is a risk factor in the distribution of ESBL producers (Najjuka et al., 2016; Xiao et al., 2019). In the present study, the prevalence of ESBL-producing isolates (*E. coli* and *K. pneumoniae*) was found more in males than females. The results are comparable to the studies done in Iran (Peerayeh et al., 2016) and elsewhere (Ouedraogo et al., 2016), but differ from other investigations that revealed that a high prevalence of ESBL producers detected in female patients (Chander and Shrestha, 2013; Abayneh et al., 2018; Kettani Halabi et al., 2021). The reason for these differences is not clear. Urinary and respiratory tract colonization by ESBL-producing bacteria in male patients could be responsible. However, there are no existing studies to explain this speculation. In this study, the highest ESBL-producing isolates (*E. coli* and *K. pneumoniae*) were detected in individuals aged over 60 years. Several investigations support that advanced age (mostly over 60 years) is a risk factor for severe infection with ESBL producers. (Hongsuwan et al., 2014; Heytens et al., 2017; Kettani Halabi et al., 2021). One potential reason for this is that old age is associated with poor health and status. Other age groups, including those under 60 years are not spared from this risk. (Halabi et al., 2021; Heytens et al., 2017). Our study observed the highest infection rates of ESBL-producers among patients in medical units. This is not surprising because the medical wards had a higher number of hospitalized patients during the study period. Patients in these wards usually have indwelling devices and extended hospital stays. These two features are well-defined risk factors for the widespread distribution of ESBL-producing pathogens among individuals admitted to hospitals (Lautenbach et al., 2001; Apisarnthanarak et al., 2007). Recently, types of clinical samples are considered as contributing factors to the spread of ESBL-positive strains (Ouedraogo et al., 2016). A high proportion of ESBL-positive *E. coli* strains was found in urine samples in this study, similar to a previous report by Hassuna et al. (2020), who noted that approximately 59.7% of uropathogenic *E. coli* were ESBL producers. On the other hand, most ESBL-producing *K. pneumoniae* isolates appeared in sputum specimens, which was consistent with another study in Taiwan (Cheng et al., 2016) and elsewhere (Caron et al., 2018). The predominance of these two species in urine and sputum samples may be due to their colonization as normal bacterial flora in the urinary (mainly *E. coli*) and respiratory tract (particularly *K. pneumoniae*) (Obeng-Nkrumah et al., 2013; Ouedraogo et al., 2016). The resistance profiles of ESBL-positive and ESBL-negative strains (*E. coli* and *K. pneumoniae*) differed widely based on the class of antibacterial drugs used.In our study, non-ESBL-producing isolates showed high resistant rates of 72.2% (*E. coli*) and 100% (*K. pneumoniae*) to ampicillin. Similarly, other investigations reported that a resistance rate of 100% to ampicillin was observed for non-ESBL-producing bacteria (*E. coli* and *K. pneumoniae*) (Seni et al., 2016; Abayneh et al., 2018). It is possible that these isolates may possess strategies for antibiotic resistance that include the expression of ampC lactamase and metallo-bata-lactamase (Dalela et al., 2012). These findings probably reflect the improper prevention and control of the transmission of antibiotic-resistant bacteria, and few therapeutic options will remain for individuals infected with these pathogen shortly.

All ESBL-positive *E. coli* strains (99.8%) were resistant to ampicillin, similar to the resistance rate of ESBL-positive *K. pneumoniae* strains (100%). Our results agree with another study conducted in Ethiopia (Abayneh et al., 2018). Notably, penicillin combined with a beta-lactamase inhibitor such as piperacillin/tazobactam was demonstrated to be effective against ESBL producers (resistance rates of 8.5% and 27.9% in *E. coli* and *K. pneumoniae*, respectively). Conversely, other studies documented that 40% (Kettani Halabi et al., 2021) and 74.5% (Najjuka et al., 2016) of ESBL-producing pathogens were resistant to this antimicrobial agent. In this study, some third-generation cephalosporins exhibited low effectiveness against ESBL-positive pathogens. Resistance rates of ESBL-producing *E. coli* isolates were 100% and 71.3% for cefotaxime and ceftazidime, respectively. In comparison, ESBL-positive *K. pneumoniae* strains revealed similar resistance rates to cefotaxime (100%) and ceftazidime (85.4%). These data are consistent with previous studies conducted in China (Quan et al., 2017), Nepal (Chander et al., 2013), Ethiopia (Abayneh et al., 2018) and Morocco (Kettani Halabi et al., 2021). In addition, cefoperazone/sulbactam, a third-generation cephamycin beta-lactamase inhibitor combination, had antibacterial potential against ESBL-producing isolates (resistance rates of 10.7% and 43.3% in *E. coli* and *K. pneumoniae*, respectively). Similarly, other reports noted that ESBL producers showed low resistance rates of 8.1–12.3% (*E. coli*) and 16.1% (*K. pneumoniae*) to cefoperazone/sulbactam (Yang et al., 2015; Jia et al., 2021). Carbapenems exhibited high potential against all ESBL-positive strains in this study, with a susceptibility rate of 100%, which is similar to that recorded in other studies (Abayneh et al., 2018; Chander et al., 2013). Our study supports the conclusion that carbapenems are the first-line drug of choice for severe infections with ESBL-producing bacteria.

We found that ESBL-producing isolates showed high resistance varying from 53.8% to 76.6% to quinolones, ciprofloxacin, and norfloxacin. These data parallel a previous study by Abayneh et al. (2018), who demonstrated that approximately 76.5% of ESBL producers were resistant to these drug classes. A minority of ESBL-positive isolates were resistant to amikacin (*E. coli*: 1.9%; *K. pneumoniae*: 3.8%), while moderate bacterial resistance to another aminoglycoside, gentamicin, was observed (*E. coli*: 52%; *K. pneumoniae*: 36.4%). This result is consistent with a previous study conducted in Nepal, where resistance rates of 6.6% and 12.5% to amikacin were observed for ESBL-positive *E. coli* and *K. pneumoniae*, respectively. At the same time, 23.3% and 45.3% of *E. coli* and *K. pneumoniae* strains, respectively, were resistant to gentamicin(Chander et al., 2013). Thus, aminoglycosides could be the drugs of choice for patients infected with ESBL producers. The resistance rate of ESBL-producing *E. coli* isolates to trimethoprim/sulfamethoxazole (68.7%) in our study was consistent with other studies done in Ethiopia (65.1%) and Tanzania (76%) (Blomberg et al., 2004; Kibret and Abera, 2011). For *K. pneumoniae*, the highest resistance rate was recorded against trimethoprim/sulfamethoxazole (79.3%). These results were also similar to studies performed in Ethiopia (86.4%) and Iran (91.4%) (Mansouri and Abbasi, 2010; Teklu et al., 2019). This study showed an increase in the prevalence of ESBL-producing *Enterobacterales* and their high resistance rate to many families of antibiotics in Chiangrai Prachanukroh Hospital, probably reflecting the overuse or misuse of antibiotics combined with unreliable diagnostic practices in resource-limited settings.

Our work also has some limitations. First, this retrospective study was conducted in a single center at a tertiary hospital in Chiang-Rai province, northern Thailand. Therefore, the high prevalence of ESBL producers found in this study does not accurately represent the prevalence of ESBL-producing isolates in the overall population of individuals admitted to hospitals in Chiang-Rai. We recommend that multicenter surveillance be conducted in the future to overcome the existing factor of these limitations in our area. Second, the lack of data on bacterial cultures due to the loss of some clinical specimens also limited our ability to assess the incidence of ESBL producers among hospitalized patients. Third, this study performed the combined disk test instead of the E-test strip (golden method) for the confirmation of ESBL producers. The reason for this is that the combination of disk tests is simple, inexpensive, and convenient for our routine microbiology laboratory. Although this method had a sensitivity of 100% for ESBL detection, but poorly specific. (Jacoby et al., 2006; Polsfuss et al., 2012). Finally, advanced molecular analysis for species classification and ESBL typing were not performed in our study. Thus, it is possible that some ESBL-producing isolates in this study were incorrectly identified and mistakenly counted. Therefore, a more sophisticated procedure for identifying ESBL producers should be available for future investigations.

## Conclusion

Our study demonstrated a high prevalence of ESBL-producing *Enterobacterales* among patients in a tertiary hospital in the north of Thailand. ESBL producers were characterized primarily as being *E. coli* strains rather than *K. pneumoniae*. A high proportion of ESBL-positive isolates was detected in patients aged over 60 years and in men.ESBL-producing *E. coli* and *K. pneumoniae* isolates were predominantly found in urine and sputum specimens, respectively. This study revealed an increase in resistance to many classes of antibacterial drugs among both ESBL-positive and ESBL-negative isolates. However, carbapenems and aminoglycosides showed potential against ESBL producers. Hence, these two drugs are a good choice for treating infections of ESBL-producing microorganisms.

## Data availability statement

The original contributions presented in the study are included in the article/supplementary material. Further inquiries can be directed to the corresponding author.

## Ethics statement

The studies involving human participants were reviewed and approved by the University of Phayao Human Ethics Committee. Written informed consent for participation was not required for this work in accordance with national legislation and institutional requirements.

## Author contributions

Study design: AS, TK, and KK. Data collection: AS, TK, CB, AK, CT, ChoT, NS, and KK. Data interpretation: AS, TK, CT, NS, and KK. Statistical analysis: AS, CT, and KK. All authors contributed to the initial draft of the manuscript. AS, NS, and KK approved the submitted version.

## Funding

This study was partially supported by grant FF65-RIM11 from the University of Phayao, Thailand, and by the School of Medical Sciences, University of Phayao (MS 201003).

## Conflict of interest

The authors declare that the research was conducted in the absence of any commercial or financial relationships that could be construed as a potential conflict of interest.

## Publisher’s note

All claims expressed in this article are solely those of the authors and do not necessarily represent those of their affiliated organizations, or those of the publisher, the editors and the reviewers. Any product that may be evaluated in this article, or claim that may be made by its manufacturer, is not guaranteed or endorsed by the publisher.
